# Characteristics of Heat Illness during Hajj: A Cross-Sectional Study

**DOI:** 10.1155/2018/5629474

**Published:** 2018-02-14

**Authors:** Doaa A. Abdelmoety, Nahid K. El-Bakri, Wedian O. Almowalld, Zyad A. Turkistani, Bassam H. Bugis, Eshraq A. Baseif, Mohamed H. Melbari, Khaled AlHarbi, Amani Abu-Shaheen

**Affiliations:** ^1^Clinical Research Management Department, Executive Administration of Research, King Abdullah Medical City in Holy Capital, Makkah, Saudi Arabia; ^2^Research Center, King Fahad Medical City, Riyadh, Saudi Arabia; ^3^Medical College, Umm Al-Qura University, Al Taif Road, Makkah 24382, Saudi Arabia; ^4^Planning and Research Department, General Directorate of Health Affairs, Makkah, Saudi Arabia; ^5^Emergency Hospital At Mina, Makkah 24243, Saudi Arabia

## Abstract

**Introduction:**

Data regarding the characteristics and outcomes of heat illness are lacking in the literature. The present study aimed to identify the clinical characteristics, morbidity, management, and mortality associated with heat illness among Hajj participants.

**Methods:**

A cross-sectional study was conducted during the Hajj in 2016 on patients who presented to emergency departments and were diagnosed with heat exhaustion or heatstroke. Data were collected using a structured collection sheet developed based on the literature.

**Results:**

A total of 267 patients were recruited. Of these, 80 (29%) and 187 (67.75%) were diagnosed with heatstroke and heat exhaustion, with 6.3% and 0.0% mortality, respectively. The mean age of the patients was 54.0 ± 16 years. Diabetes mellitus was the most common comorbidity among both heatstroke and heat exhaustion patients. The majority of patients had hyperthermia and electrolyte imbalance. Most of the heat illness cases were treated per heat illness guidelines.

**Conclusions:**

Although authorities are working on research and forming interdisciplinary teams to prevent health problems during the Hajj, the mortality rate from heatstroke is high and the majority of the patients had hyperthermia, varied signs and symptoms, elevated creatinine levels, and electrolyte imbalances.

## 1. Introduction

The average global temperature has been estimated to increase in the summer months by 0.6°C per century [1]. Heat-related illness results in at least 70,000 fatalities annually in Europe [2] and more than 1,300 deaths per year in the United States [3]. High temperatures during the summer season are associated with heat illness, which can range from minor (heat cramps, edema, prickly heat, and syncope) to serious conditions (heat exhaustion or heatstroke) [4]. Many factors play a role in the outcome of heat illness, including old age, chronic disease, overcrowding, physical exertion, lack of acclimatization, and dehydration. A lack of proper treatment could exacerbate the condition to organ failure, convulsions, coma, and increasing intracranial pressure [5, 6]. Accordingly, Hajj, an Islamic pilgrimage, is one of the largest mass gathering challenges for the Kingdom of Saudi Arabia and the Ministry of Health. Hajj is the fifth pillar of Islam and it is mandatory for Muslims to perform Hajj at least once in their lifetime if they are financially and physically capable. It attracts more than 2.5 million pilgrims from many countries, who assemble in Makkah over a 1-week period [7]. Depending on the Islamic (lunar) calendar, Hajj may occur during the summer season, when the temperature can exceed 45°C.

The Saudi Ministry of Health, in collaboration with the Hajj Committees, prepares for emergencies during Hajj as well as for communicable and noncommunicable diseases [8] by providing the necessary equipment, facilities, and trained staff to hospitals in Mina, El-Mashaeer, and Makkah [9–11].

Previous studies have investigated different aspects of heat illness in Saudi Arabia, including the epidemiology [12] and environmental conditions [13] by evaluating changes in climatic heat, and heat illness as a noncommunicable disease [8, 10, 11]. Moreover, studies are lacking in the literature regarding disease course and patient characteristics, treatments, outcomes, and complications of heat illness during Hajj [14]. Earlier studies have focused on the variables and clinical information that affect medical needs during Hajj for a specific hospital or area [8, 15–17]. However, a systematic approach to data collection that includes multiple hospitals and areas with a focus on heat illness has not been performed. Therefore, the aim of the present study was to identify the type, clinical characteristics, risk factors, laboratory profiles, morbidity, medical management, and mortality associated with heat illness among Hajj participants. The results of this study might provide information on the hazards to be expected during the summer season and offer guidance to Hajj authorities.

## 2. Materials and Methods

### 2.1. Study Design

A prospective cross-sectional study was conducted during the Hajj in 2016 at four hospitals in the Mena region (Mena Emergency Hospital, Mena Alwadi Hospital, Mena Alshareih Algadeid Hospital, and Mena Aljassir Hospital) and four hospitals in the Arafat region (Namera Hospital, Jabal Elrahmah Hospital, East Arafat Hospital, and Arafat General Hospital) of Saudi Arabia.

### 2.2. Participants

Patients who presented to the emergency departments of any of the eight hospitals and who were diagnosed with heat exhaustion or heatstroke were included in this study. Heat exhaustion was defined as mild-to-moderate heat-related illness owing to the exposure to high environmental heat; the signs and symptoms included intense thirst, weakness, discomfort, anxiety, dizziness, and syncope; the temperature could be normal or slightly elevated, >37°C (98.6°F), but less than 40°C (104°F). Heatstroke was defined as a severe heat-related illness characterized by a core temperature >40°C (104°F) and central nervous system abnormalities resulting from passive exposure to environmental heat [18].

### 2.3. Data Collection

Data were collected through a structured collection sheet which was developed through an in-depth review of the literature [18]. The data collection sheet included information ascertaining: (i) patient clinical characteristics (vital signs, comorbidities, and signs and symptoms), (ii) laboratory profiles (blood gas, electrolytes, liver and kidney enzymes, hemoglobin, platelets, and glucose), (iii) medical management (ensuring airway patency, rehydration, cooling, and management of complications if any), and (iv) outcomes (improved and discharged, refused treatment, transferred to another hospital, discharged against medical advice, or died). Demographic data, including patient age, nationality, Hajj or non-Hajj participant, and gender, were also collected.

### 2.4. Ethical Considerations

The Institutional Review Board of King Abdullah Medical City exempted the study protocol from obtaining ethical approval.

### 2.5. Statistical Analysis

Data were analyzed using IBM SPSS Statistics for Windows, version 22.0 (IBM Corp., Armonk, NY, USA). Numeric data were presented as means ± standard deviations (SDs) or medians and interquartile ranges (IQRs) according to the type of distribution, while categorical variables were presented as percentages. *t*-test and Chi-square and Fisher's exact tests were used as appropriate.

## 3. Results

### 3.1. Demographic Characteristics

During the study period, 267 patients were recruited. Of these, 80 (29%) and 187 (67.75%) were diagnosed with heatstroke and heat exhaustion, respectively. The mean age of the patients was 54.0 ± 16 years (median: 58 years, range: 20–100 years). A total of 143 (54.6%) patients were male. The patients were residents of 30 different countries. Most patients were from India (16.7%), Egypt (16.7%), Indonesia (11.1%), Saudi Arabia (8.9%), and Pakistan (8.5%), and 38.1% were of other nationalities. Mina Alwadi and Mina Emergency Hospitals received the most number of patients with heatstroke (29 [36.3%] and 16 [20.0%] patients, resp.). Mina Aljassir Hospital received the most number of heat exhaustion cases (71 [38.0%] patients).

The mean ages of the patients with heatstroke and heat exhaustion were 57.41 ± 12.35 and 52.49 ± 17.70 years, respectively. The majority of patients in both groups were male (58.7% and 52.4%, resp.).

### 3.2. Clinical Characteristics

#### 3.2.1. Comorbidities

Among the patients with heatstroke, 15.0% had diabetes; however, only 5.9% of patients with heat exhaustion had diabetes (*p* = 0.029) (Table 1).

#### 3.2.2. Vital Signs

Patients with heat exhaustion and with heatstroke had recorded temperatures of <40°C and >40°C, respectively. The vital signs of the two groups are presented in Table 2.

#### 3.2.3. Signs and Symptoms

Altered mental status (50 patients, 62.5%), tachycardia (37 patients, 46.3%), and tachypnea (29 patients, 36.3%) were the most common signs and symptoms among patients with heatstroke. Dizziness (40 patients, 21.4%) and vomiting (20 patients, 10.7%) were the most common signs and symptoms among patients with heat exhaustion (Table 3). However, a statistically significant difference was only found between heatstroke, heat exhaustion, and irritability (*p* = 0.028).

### 3.3. Laboratory Results

Physicians requested blood gas, clinical chemistry, and complete blood profiles for some patients according to their medical conditions. Among those with heatstroke, the blood gas investigations indicated partially compensated metabolic acidosis with hypoxemia in 26 (33%) patients. All blood gas values were normal in patients with heat exhaustion, except for decreased oxygen partial pressure (pO_2_) values in 24 (19%) patients, indicating hypoxemia.

The top four abnormal results among patients with heatstroke were high glucose level (36 patients, 45.0%), high creatinine concentration (33 patients, 41.3%), hyponatremia (21 patients, 26.3%), and high blood urea nitrogen (BUN) concentration (21 patients, 26.3%). In comparison, hyponatremia (18 patients, 9.6%), hypokalemia (18 patients, 9.6%), high glucose level (15 patients, 8.5%), and high BUN concentration (15 patients, 8.0%) were the top abnormal results among patients with heat exhaustion (Table 4).

Statistical significant difference was found between heatstroke, heat exhaustion, and the following laboratory tests: high glucose level (*p* = 0.004), high creatinine (*p* = <0.001), hyponatremia (*p* = 0.046), high BUN (*p* = 0.023), hypokalemia (*p* = 0.045), low platelets (PLT) (*p* = 0.003), low hemoglobin (Hb) (*p* = 0.037), and high white blood cell (CK) (*p* = 0.044).

### 3.4. Management and Outcomes

Measures to ensure airway patency and intubation were provided to 51 (63.8%) heatstroke and 9 (4.8%) heat exhaustion patients. Rehydration by intravenous line, cardiac monitor, and Foley catheter were provided to 64 (80%) heatstroke and 20 (10.7%) heat exhaustion patients. Most of the patients with heat illness were managed with the use of a fan, water spray, and ice packs to reduce the body temperature. The median length of the hospital stay was two hours (IQR, 1–12 hours), with a maximum stay of 57 hours. Around 84.3% of patients with heatstroke improved and were discharged; however, 7.1% died and 5.7% were admitted to critical care units. Of the 178 patients with heat exhaustion, 162 (94.7%) improved and were discharged, 7 (4.1%) discharged against medical advice, 2 (1.2%) were admitted to critical care units, and none died (*p* = 0.001) (Table 5).

## 4. Discussion

The results of the present study provide extensive data regarding the types, clinical characteristics, laboratory profiles, morbidity, medical management, and mortality due to heat illness among pilgrims. Heat illness can be prevented with strategic planning and preventive measurements. A 2012 systematic review of weather and environmental hazards at mass gatherings showed that a one-degree increase in temperature, from 20°C to 21°C, resulted in an 11% increase in the number of individuals requiring medical attention. Moreover, temperatures exceeding 27°C resulted in an increase in the incidence of patient presentations [19]. Thus, the government of Kingdom of Saudi Arabia, in collaboration with the Ministry of Hajj, is taking measures to prevent health problems during the event [20]. During Hajj, participants are educated to stay in shelters for protection, use sunblock [21], perform proper handwashing, and ensure adequate water intake [22]. Also, participants are encouraged to be flexible with time, if possible, and perform rites during the night [21]. However, the results of the present study showed that out of 267 patients 80 (29%) patients had heatstroke, the most severe form of heat illnesses, with mortality of 6.3%.

In our study, the mean age of the patients was 54.0 ± 16 years, with a predominant proportion of males. Moreover, diabetes mellitus was the most common comorbidity among both heatstroke and heat exhaustion patients. Previous epidemiological studies have shown that the elderly and those with comorbidities have an increased risk of hospitalization due to heatstroke [23, 24].

Elderly people are susceptible to heat illness due to decreased blood flow to the skin, sweat gland function, cardiac output, thirst sensation, and renal function [25, 26]. Takahiro et al. reported age ≥ 65 years, elevated body temperature, deterioration of consciousness, and high serum creatinine level to be the predictive factors of patient hospitalization [27]. Therefore, these factors can be used to identify a potentially severe condition in the early stage of illness before the condition worsens.

Since the central nervous system is sensitive to heat stress [28, 29], its dysfunction is a major symptom of hyperthermia [23, 29]. In early period of heat illness, the common symptoms include anxiety, dizziness, fainting, and headache [5]. With progression to a pathological condition that leads to decreased cerebral blood flow and increased intracranial pressure, patients commonly experience delirium, convulsions, and coma [6, 28]. Similarly, our study showed that patients with heatstroke had hyperthermia, altered mental status, tachycardia, convulsions, high creatinine level, and electrolyte imbalances. To prevent the unfavorable consequences of a delay in starting treatment, measurement of body temperature and consciousness level are simple but important indicators of the severity of heat illness.

Consistent with our findings regarding patient management of these illnesses, previous reports showed that Hajj participants were managed according to heat illness guidelines [4, 30] recommending cooling, fluid repletion, and blood tests to exclude organ damage, according to patient conditions. Preventive measures and awareness about the dangers of dehydration, as well as encouraging Hajj participants to drink sufficient fluids, seek shade, and recognize the symptoms of dehydration, may reduce the incidence of heat illness. Similar measures have been reported to decrease the incidence of heat illness in other mass gatherings [31].

Hajj authorities are working on research, education, and the formation of interdisciplinary teams comprising environment specialists and health experts [30]. Studies on Hajj in the past decade have encouraged research and planning [21], which led to a massive reduction in the incidences of communicable diseases that were major causes of mortality [15, 16]. However, noncommunicable diseases remain an issue, especially for individuals with comorbidities, those in older age groups, and those living in high-temperature locations [15, 17]. Correspondingly, our results revealed a high rate of mortality from heatstroke.

Hajj is a major annual mass gathering of millions of people from all over the world, with different cultures, socioeconomic levels, knowledge, attitudes, and, most importantly, health statuses [30]. The causes of morbidities and mortalities among such a broad spectrum of people are varied [15, 21, 32]. However, with proper education and awareness measures, most of these causes can be reduced.

The current findings might provide a foundation for strategic planners to make better arrangements for Hajj participants during the summer season. Furthermore, our results confirm the importance of screening individuals in their home countries before Hajj based on selected criteria to exclude unfit persons from participating or to provide a fast-track process during Hajj.

Future interventional studies should highlight the importance of heat illness education and prevention.

## 5. Conclusion and Recommendations

The present study documented the demographic and clinical characteristics, mortality, and medical management of patients with serious heat illness, which may help to identify patients with mild-to-moderate heat illness who should be observed in emergency departments. Old age and diabetes mellitus were the most common risk factors for heat illness. Although authorities are working on research, education, and the formation of interdisciplinary teams comprising environment specialists and health experts and taking measures to prevent health problems during Hajj, the mortality rate due to heatstroke requires attention. In addition, the majority of patients had hyperthermia, varied signs and symptoms, elevated creatinine levels, and electrolyte imbalances. Increased patient education and awareness regarding heat illnesses may reduce the occurrence and consequences of these heat-related symptoms and illnesses.

## Figures and Tables

**Table 1:** Comorbidities of patients with heatstroke and heat exhaustion.

Variable	Heatstroke *n* = 80	Heat exhaustion *n* = 187	*p* value
Cardiovascular disease			
Yes	2 (2.5)	4 (2.2)	1.000
No	78 (97.5)	183 (97.8)	
Diabetes mellitus			
Yes	12 (15.0)	11 (5.9)	0.029^*∗*^
No	68 (85.0)	176 (94.1)	
Hypertension			
Yes	5 (6.3)	8 (4.3)	0.539
No	75 (93.7)	179 (95.7)	

Data presented as numbers (percentages). ^*∗*^*p* value is statistically significant.

**Table 2:** Vital signs of patients with heatstroke and heat exhaustion.

Vital signs	Heatstroke *n* = 80	Heat exhaustion *n* = 187	*p* value
	Mean ± SD	Mean ± SD	
Systolic blood pressure (mm Hg)	126 ± 24	123 ± 20	0.493
Diastolic blood pressure (mm Hg)	76 ± 17	72 ± 13	0.177
Respiratory rate (breaths/minute)	25 ± 14	30 ± 24	0.454
Pulse rate (beats/minute)	110 ± 21	98 ± 18	0.003^*∗*^

^*∗*^ *p* value is statistically significant.

**Table 3:** Signs and symptoms of patients with heatstroke and heat exhaustion.

Vital signs	Heatstroke *n* (%)	Heat exhaustion *n* (%)	*p* value
Altered mental status	50 (62.5)	7 (4)	0.315
Tachycardia	37 (46.3)	7 (4)	0.285
Tachypnea	29 (36.3)	4 (2.1)	0.422
Hypotension	19 (23.8)	3 (1.6)	0.474
Convulsions	11 (13.8)	1 (0.5)	0.693
Diarrhea	5 (6.3)	1 (0.5)	0.337
Dizziness	1 (1.2)	40 (21.4)	0.412
Vomiting	0	20 (10.7)	0.052
Headache	0	14 (7.5)	0.061
Irritability	1 (1.2)	12 (6.4)	**0.028** ^**∗**^
Nausea	0	6 (3.2)	0.058
Postural hypotension	0	4 (2.1)	0.099

^*∗*^ *p* value is statistically significant.

**Table 4:** Laboratory profiles of the study's participants.

Test	Heatstroke *n* (%)	Heat exhaustion *n* (%)	*p* value
High glucose level	36 (45.0)	15 (8.5)	**0.004** ^**∗**^
High creatinine	33 (41.3)	9 (4.8)	**<0.001** ^**∗**^
Hyponatremia	21 (26.3)	18 (9.6)	**0.046** ^**∗**^
High BUN	21 (26.3)	15 (8.0)	**0.023** ^**∗**^
Hypokalemia	20 (25.0)	18 (9.6)	**0.045** ^**∗**^
Low PLT	17 (21.3)	8 (4.3)	**0.003** ^**∗**^
Low Hb	16 (20.0)	13 (7.0)	**0.037** ^**∗**^
High WBC	13 (16.2)	12 (6.4)	0.610
High CK	13 (16.3)	8 (4.3)	**0.044** ^**∗**^
High AST	10 (12.5)	10 (5.3)	1.000
High LDH	8 (10)	4 (2.1)	0.165
High ALT	2 (2.5)	5 (2.7)	0.390

BUN: blood urea nitrogen; PLT: platelets; Hb: hemoglobin; WBC: white blood cell; CK: creatinine kinase; AST: aspartate transaminase; LDH: lactate dehydrogenase; ALT: alanine transaminase. ^*∗*^*p* value is statistically significant.

**Table 5:** Outcomes of patients with heatstroke and heat exhaustion.

	Heatstroke *n* (%)	Heat exhaustion *n* (%)	*p* value
Improved and discharged	59 (84.3)	162 (94.7)	**0.001** ^**∗**^
Died	5 (7.1)	0	
Admitted to critical care units	4 (5.7)	2 (1.2)	
Transferred to another hospital	1 (1.4)	0	
Discharged against medical advice	1 (1.4)	7 (4.1)	

^*∗*^ *p* value is statistically significant.
